# High Titers of Low Affinity Antibodies in COVID-19 Patients Are Associated With Disease Severity

**DOI:** 10.3389/fimmu.2022.867716

**Published:** 2022-04-13

**Authors:** Jan Hendriks, Richard Schasfoort, Michelle Koerselman, Maureen Dannenberg, Alexander Daniel Cornet, Albertus Beishuizen, Job van der Palen, Johannes Krabbe, Alide H. L. Mulder, Marcel Karperien

**Affiliations:** ^1^ Department of Developmental BioEngineering, Faculty of Science and Technology, University of Twente, Enschede, Netherlands; ^2^ Department of Medical Cell BioPhysics, Faculty of Science and Technology, University of Twente, Enschede, Netherlands; ^3^ Intensive Care Center, Medisch Spectrum Twente, Enschede, Netherlands; ^4^ Medical School, Medisch Spectrum Twente, Enschede, Netherlands; ^5^ Section Cognition, Education and Data, Faculty of Behavioural, Management and Social Sciences, University of Twente, Enschede, Netherlands; ^6^ Department of Clinical Chemistry, Medlon BV, Enschede, Netherlands; ^7^ Department of Clinical Chemistry and Laboratory Medicine, Medisch Spectrum Twente, Enschede, Netherlands; ^8^ Department of Clinical Chemistry, Ziekenhuis Groep Twente, Almelo, Netherlands

**Keywords:** COVID-19, viral infection, antibodies, immunoassay, infectious diseases, SPRi (surface plasmon resonance imagery)

## Abstract

**Background:**

Almost 2 years from the beginning of the coronavirus disease 2019 (COVID-19) pandemic, there is still a lot unknown how the humoral response affects disease progression. In this study, we investigated humoral antibody responses against specific SARS-CoV2 proteins, their strength of binding, and their relationship with COVID severity and clinical information. Furthermore, we studied the interactions of the specific receptor-binding domain (RBD) in more depth by characterizing specific antibody response to a peptide library.

**Materials and Methods:**

We measured specific antibodies of isotypes IgM, IgG, and IgA, as well as their binding strength against the SARS-CoV2 antigens RBD, NCP, S1, and S1S2 in sera of 76 COVID-19 patients using surface plasmon resonance imaging. In addition, these samples were analyzed using a peptide epitope mapping assay, which consists of a library of peptides originating from the RBD.

**Results:**

A positive association was observed between disease severity and IgG antibody titers against all SARS-CoV2 proteins and additionally for IgM and IgA antibodies directed against RBD. Interestingly, in contrast to the titer of antibodies, the binding strength went down with increasing disease severity. Within the critically ill patient group, a positive association with pulmonary embolism, d-dimer, and antibody titers was observed.

**Conclusion:**

In critically ill patients, antibody production is high, but affinity is low, and maturation is impaired. This may play a role in disease exacerbation and could be valuable as a prognostic marker for predicting severity.

## Introduction

The coronavirus disease 2019 (COVID-19) pandemic has disrupted global society, critically stressed healthcare systems, and resulted in a relatively high mortality and morbidity with continued high need for patient care (1). Even after 1½ years of intensive international (scientific) effort, many questions remain about the underlying pathology (2), distinctive patient factors determining severity (3), and on the protective or damaging role of humoral immune system (4).

A striking characteristic of COVID-19 is the large heterogeneity in patient response to the viral infection (5). A large group of patients suffers mild or even asymptomatic disease (6), but in a smaller group, the infection progresses and escalates, resulting in hospitalization and potential death (7). Clear risk factors are (old) age, gender, obesity, and underlying morbidities (8). However, how associated immune characteristics contribute to this susceptibility is still largely unknown (3, 9).

The immune system is heavily involved in battling the virus, and patients with COVID-19 generally develop strong cellular and humoral responses (10). Within 1 to 2 weeks, increasing antibody titers are found in most patients, regardless of disease severity (11), with neutralizing capacity (12, 13). However, disease severity generally does not quickly resolve as a result of the presence of these neutralizing antibodies (13, 14). In fact, there is emerging evidence of a potential deleterious role of the humoral response in the severity of the disease (15). Therefore, there appears to be a delicate balance of a protective effect and hyperreaction of the immune system leading to organ/tissue damage and potential death (16, 17).

Yet, the factors determining the balance between disease attenuation and disease amplification are not well understood (11). For example, most articles focus on single viral proteins [in particular spike (S) or nucleocapsid (NCP)]. Therefore, there is limited information on the dynamics of the antibody response toward specific viral targets [e.g., receptor-binding domain (RBD), S1, S2, or NCP] and the ratio of the response to these viral proteins. Moreover, several studies contradict each other regarding the longitudinal trends in antibody production (13, 18) and titer in mild versus severe disease (19). These limitations and contradictory results are partly caused by the lack of consistency in comparison groups, study design, the heterogeneity of assays that were used, and by indirect antibody measurements.

Antibody measurements are typically performed using enzyme-linked immunosorbent assay (ELISA) or related immunoassays. In addition to relative long assay times, testing of IgM, IgG, and IgA isotopes requires individual assays. Furthermore, standard immunoassays provide only indirect information on antibody kinetics and affinities. An attractive alternative is surface plasmon resonance imaging (SPRi). In previous studies, we have demonstrated a high-throughput SPRi assay for the quantitative measurement of IgM, IgG, and IgA antibodies and their apparent polyclonal affinity in the sera of COVID-19 patients in one single experiment with a run time of less than 30 min (20, 21). This method is ideally suitable for measuring concentrations of antibodies in patients as well as determining their strength of binding.

In this article, we applied the high-throughput SPRi assay for a detailed and comprehensive characterization of patient humoral response and study its relationship with COVID severity. With this approach, we measured total antibody response and concentrations of specific IgG, IgM, and IgA isotypes against the most important SARS-CoV2 viral proteins, that is, RBD, NCP, S1, and S1S2. We subsequently determined the affinity of these antibodies toward the individual proteins. Finally, based on the RBD sequence, we developed a peptide epitope mapping assay on SPRi to characterize antibody responses in more detail. The results of the antibody and peptide experiments were compared with clinical information, such as disease severity and patient characteristics.

## Materials and Methods

### Patient and Control Serum Samples

Residual serum samples were obtained from 76 unique COVID-19 patients confirmed by reverse transcriptase–quantitative polymerase chain reaction and computed tomography (CT) scans and collected from March to December 2020. Eight samples from cases were collected within 10 days after the first symptoms (range, 5–9 days); 68 were collected 10 or more days after the first symptoms (range, 10–70 days).

Disease severity of the SARS-CoV2 infection was classified according to the World Health Organization criteria (22) as either mild, moderate, severe, or critical. Patients with mild disease severity did not show abnormal CT imaging. Moderate patients had fever and/or classical respiratory symptoms and typical CT images of viral pneumonia. Severe patients met at least one of the following additional conditions: (1) shortness of breath with respiratory rate ≥30 times/min, (2) oxygen saturation (Spo_2_, resting state) ≤93%, or (3) Pao_2_/Fio_2_ ≤39.9 kPa (299.3 mm Hg). Critically ill patients met at least one of the extra following conditions: (1) respiratory failure that required mechanical ventilation, (2) shock, or (3) multiple organ failure that required admission to intensive care unit (ICU). Details on included patients can be found in **Table 1**.

**Table 1:** Clinical Characteristics of Included Patients.

Demographic and Clinical Characteristics	
Age, mean ± SD, years	63 ± 14
Sex, n (%)	
Males	46 (60)
Females	30 (40)
COVID-19 severity score, n (%)	
Mild	12 (16)
Hospitalized	33 (43)
Moderate	19 (25)
Severe	14 (18)
Critical	31 (41)
ICU admission, n (%)	33 (43)
Survival, n (%)	68 (89)
Pulmonary embolism, n (%)	11 (14)
Peak d-dimer, median (range), µg/L	2,724 (<150–8,819)
Peak LDH, median (range), U/L	429 (200–842)
Peak ferritin, median (range), mg/L	1,449 (128–10,437)
Peak CRP, median (range), mg/L	217 (13–549)

### Surface Plasmon Resonance Imaging and Spotting Instruments

The IBIS MX96 instrument (IBIS Technologies) applies a valve-less consecutive injection of samples with “back-and-forth” flow [1]. The continuous flow microspotter (Wasatch Microfluidics) enables high-reliability printing of ligand molecules under flow conditions [2]. SensEye^®^ sensors (gel-type E2S, Ssens) using preactivated surface chemistry or streptavidin coated sensors were applied for printing an array of ligand samples. The instrument enables multiplex, up to 96 spots, kinetic analysis of interactions.

### Immune Response Characterization of COVID-19 Patients Using SPRi

**Figure 1:**
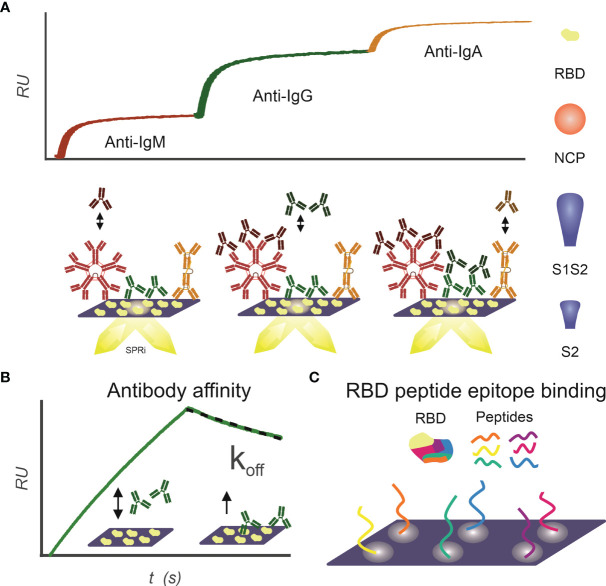
Infographics outlining the experimental design for using SPRi to characterize patient samples for the presence of SARS-CoV2 antibody responses. **(A)** IgM, IgG, and IgA antibody response toward RBD, NCP, S1S2, and S2 proteins is measured sequentially using SPRi. First patient plasma is incubated on a specific protein coupled sensor, and then anti-IgM, anti-IgG, and anti-IgA are sequentially injected, and association signal is measured in real time. **(B)** Affinity of patients’ polyclonal antibody pools toward RBD, NCP, S1S2, and S2 proteins is determined. Patient plasma is injected on protein coupled sensor, and interactions are measured in real time. The koff constant determines strength of binding and is determined in dissociation phase. **(C)** Binding epitopes of patients’ antibody pools toward RBD are determined using a 24-peptide array on SPRi.

The IgM, IgG, and IgA isotype antibody response to RBD, NCP, S1S2 (full length spike protein), and S2 antigen was determined for the included patient sera as described previously (21) (**Figure 1A**). Briefly, an SPRi sensor of a specific antigen was prepared; patient sera were spotted unto this sensor in duplicates for 3 min. In the IBIS MX96, subsequently anti-IgM, anti-IgG, and anti-IgA were injected, and maximal binding was determined (dRU).

### Multiplex Measurements to SARS-CoV2 Antigens to Determine Polyclonal Affinity

Antibody interaction affinity with SARS-CoV2 antigens was determined as described previously (21) (**Figure 1B**). Briefly, patient sera were injected under flow to a multiplex-coated SARS-CoV2 antigen sensor, with a 3-min association and 1-min dissociation time. The association rate constant *k*_a_ (abbreviated to on-rate) is the reaction rate of the antibody–antigen complex formation on the sensor surface, giving the number of complexes formed per time at unit concentration of antibody and antigen. As soon as the complex is formed on the sensor surface, dissociation of the complex can commence. The dissociation rate constant *k*_d_ (abbreviated to off-rate) expresses the number of complexes dissociating per unit of time. Equilibrium is reached, when the rates of the association and dissociation reactions are equal. When the concentration of the antibodies is equal to *K*_D_, then 50% of the molecules are bound to the ligands on the sensor surface. The association and dissociation rates of the antibodies binding to the SARS-CoV-2 proteins were calculated in Scrubber (BioLogic Software, Australia) with a 1:1 fitting algorithm. In our assay, the exact value of the dissociation constant (*k*_d_, s^−1^) for the overall binding antibodies can be determined after 30 s in the dissociation phase because the ligand density (in RU) can be measured accurately by dividing the slope with the response. This dissociation constant (*k*_d_) directly correlates to the value of the equilibrium dissociation constant [*K*_D_; Schasfoort et al. (21)].

### Epitope Mapping of Antibody Response Toward RBD

Interactions of patient sera with RBD peptides were determined to map-binding epitopes (**Figure 1C**). A peptide library of 24 peptides, 15-mer in length with 4-mer overlap of the RBD, starting from spike protein amino acid 339 to 505 was synthesized by Pepscan b.v. (peptide sequences in **Supplemental Table 1**). The peptides were biotinylated at the N-terminus using a 7-amino-3-hydroxyethyl-coumarin (AHC) linker and were spotted in duplicate on a streptavidin modified SensEye G-Strep sensor (48 × 2 spots) for 30 min. The sensor was blocked with SensEye Strep Blocking solution for 30 min in the IBIS MX96.

Patient sera were injected with 15-min association and 12-min dissociation time, after which the sensor was regenerated with a 30-s pulse of 200 mM phosphoric acid at pH 1.5. Each sensor was used for 20 patient samples, to avoid excessive sensor degradation. Positive epitopes were selected on a per-patient basis, by an automated MATLAB script (available upon request) using the criteria that interaction signal (dRU) of specific epitopes exceeds three times the standard deviation of mean of all epitopes (dRU >3 * SD).

### Statistical Analysis

Continuous variables were expressed as median and interquartile range. Nonparametric data consisting of more than two groups were analyzed using a Kruskal–Wallis test, followed by Mann–Whitney *U* test with a Bonferroni–Holm correction. Nonparametric data with two groups were analyzed using Mann–Whitney *U* test. Spearman correlation analysis was used to analyze the correlation of the different antibody specificities. A two-sided *α* of <0.05 was considered to be statistically significant. Statistical analyses were performed using OriginPro 2019b (Academic) 64-bit. Multivariate analysis was performed using SPSS software (version 28).

## Results

### Immune Response Characterization of COVID-19 Patients Using SPRi

We have developed assays to characterize the immune response using SPRi (**Figure 1**). With these assays, we measured total IgM, IgG, and IgA response simultaneously (**Figure 1A**); we measured affinity of polyclonal response (**Figure 1B**) and interaction with RBD epitopes (**Figure 1C**).

### Antibody Immune Response Toward SARS-CoV2 Antigens Per Patient Subgroup

**Figure 2:**
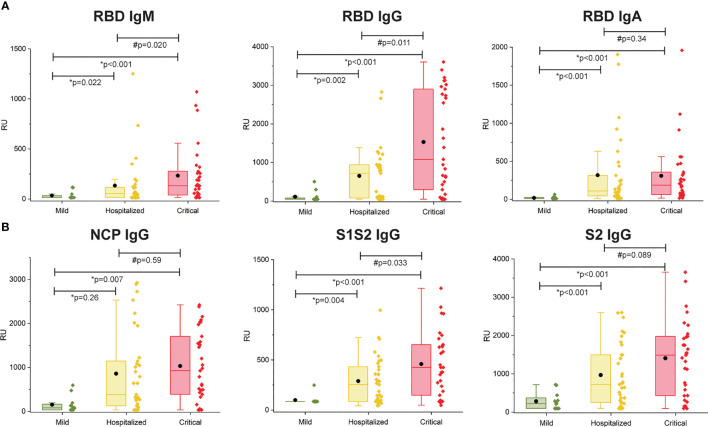
Multiplex measurement of antibody responses to four antigens of SARS-CoV2. **(A)** Total immune response (IgM, IgG, IgA) SPRi measurement of COVID-19–positive sera on RBD antigen. **(B)** IgG immune response SPRi measurement of NCP, S1S2, and S2 antigen. The boxplot represents the median, p25, and p75 values, and the black dot represents the mean SPRi RU value Comparability of groups was analyzed by Mann–Whitney *U* test. A Bonferroni–Holm procedure was used to correct for multiple comparisons between groups.

The multiplex SPRi measurement of four SARS-CoV2 antigens was used to determine the antibody immune response (IgM, IgG, IgA) per patient. The assay had good analytical sensitivity toward RBD for all isotypes (IgM, IgG, and IgA) and toward NCP, S1S2, and S2 for IgG isotype (CV <20%). Sensitivity was moderate toward NCP, S1S2, and S2 for IgM and IgA isotypes (CV >20%, CV <30%); therefore, these data were excluded from analysis. **Figure 2** shows the antibody responses stratified by mild, hospitalized, or critical disease severity. Patients with hospitalized or critical disease severity showed significantly higher responses of IgM, IgG, and IgA against RBD and IgG response against NCP, S1S2, and S2 than patients with mild disease. In addition, IgM and IgG versus RBD and IgG versus S1S2 was significantly higher in critical disease compared with patients with hospitalized disease severity. Thus, increased disease severity was positively associated with an increased titer of IgG antibodies toward all measured SARS-CoV2 antigens and with increased titer of IgM and IgA toward the RBD.

Furthermore, we found a specific subgroup of patients with critical disease with heightened RBD IgG response, with significantly increased S1S2 and S2 response (**Supplemental Figure 4**). This subgroup was characterized with a large significant increase in peak d-dimer and a lesser increase in lactate dehydrogenase (LDH) and C-reactive protein (CRP), whereas ferritin did not show a significant difference.

As expected, the titer of S1S2 IgG antibodies was correlated with the titer of S2 IgG antibodies (Spearman ρ = 0.92) and RBD IgG antibodies (Spearman ρ = 0.85). The correlation between the other antibodies was lower (S1S2 vs. NCP ρ = 0.657, S2 vs. RBD ρ = 0.781, S2 vs. NCP ρ = 0.639, and RBD vs. NCP ρ = 0.708).

### Binding Strength of the Antibodies

**Figure 3:**
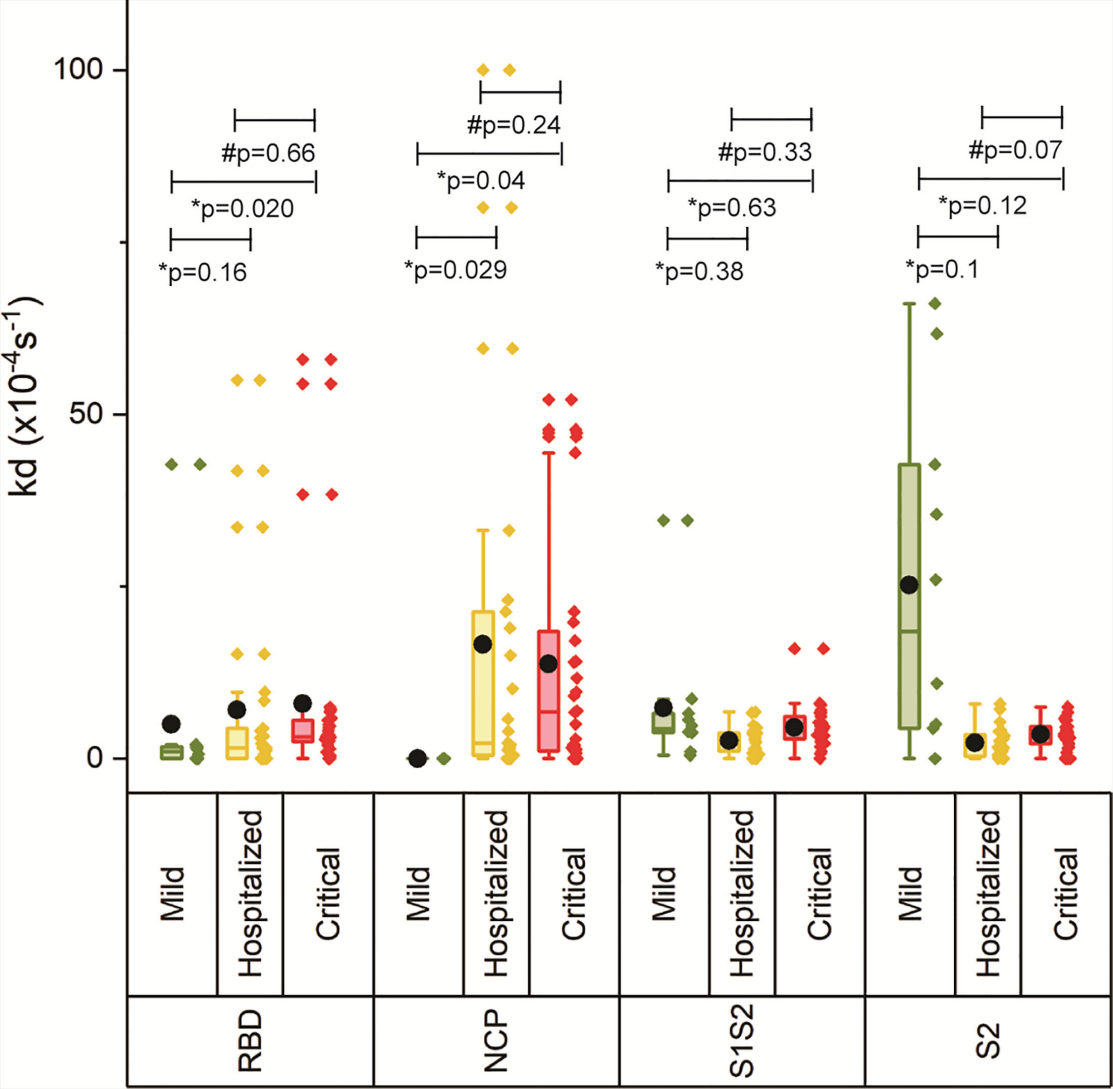
Binding strength measurements of four antigens of SARS-CoV2. The off-rate of the antibodies binding to the four antigens was determined for each severity group. The boxplot represents the median, p25, and p75 values, and the black dot represents the mean SPRi RU value. Comparability of groups was analyzed by Mann–Whitney *U* test. A Bonferroni–Holm procedure was used to correct for multiple comparisons between groups.

The off-rate (*k*_d_) was determined to rank the binding strength of the polyclonal antibody pools reacting with the respective antigen. A higher *k*_d_ correlates with a higher equilibrium dissociation constant (*K*_D_) and therefore a lower affinity (21). **Figure 3** shows increasing *k*_d_ with increasing disease severity, which was significant for critically ill patients versus mild for RBD and NCP, and critical versus hospitalized as well as mild for S1S2 and S2, suggesting decreased maturation toward antibodies with higher affinity. An exception to this is the lower measured antibody affinity toward S2, and in lesser extent toward S1S2, in mild compared with hospitalized patients and patients with critical disease. Multivariate analysis was performed on ln-transformed *k*_d_ values. Neither gender, age, body mass index, peak LDH, peak CRP, peak ferritin, IC admission, nor pulmonary embolism (PE) was attributed to the observed differences in *k*_d_ between the severity groups. The IgG response contributed significantly to the *k*_d_ of RBD, S1S2, and S2 (*p* < 0.001), whereas the *k*_d_ of NCP was unaffected by the IgG NCP. Days post onset infection (DPO) contributed also significantly to the *k*_d_ of S1S2 and S2 (*p* < 0.001) independent of the IgG response.

### Association Between Gender and Immune Response

Already since the beginning of the COVID-19 outbreak, it was observed that men were more at risk for worse outcomes and death, independent of age (2, 3). Despite this, we have found no differences between men (n = 42) and women (n = 28) regarding the antibody titers (**Supplemental Figure 1**), specificity of antibodies, or their binding strength (data not shown). Only in the group with hospitalized disease severity the anti-RBD antibody pool in men had significantly lower binding strength than in women (**Supplemental Figure 2**). This indicates that the differences in disease severity between men and women cannot be explained only by the humoral antibody response.

### Correlation between Pulmonary Embolism and SARS-CoV2 Antigens IgG Response

**Figure 4:**
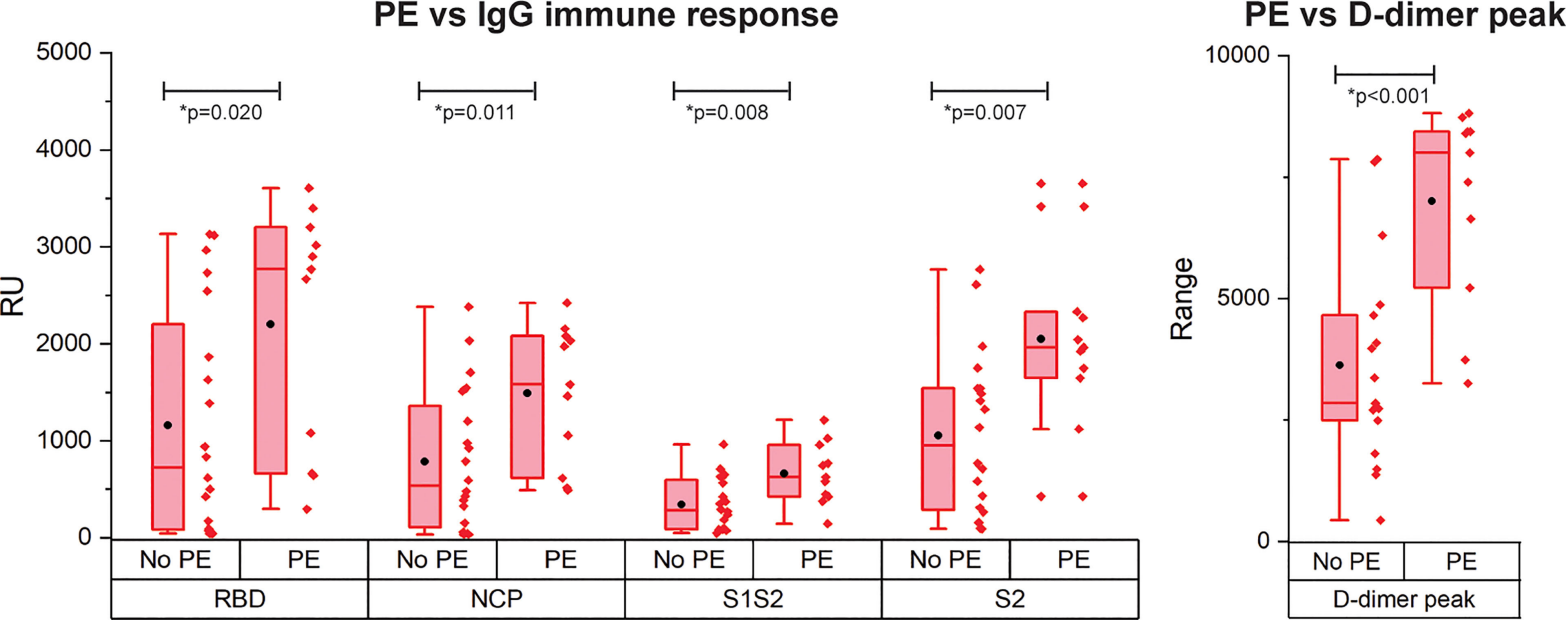
Occurrence of pulmonary embolism (PE) in IgG immune response measurement for patients with critical disease and the d-dimer peak values. N= 28 patients. The boxplot represent the median, p25 and p75 values and the black dot the mean SPRi RU value.

PE is reported frequently in COVID-19 patients and is correlated with disease severity and mortality (4). As only patients with critical disease developed PE in our cohort, we compared the immune response of patients with critical disease with and without PE (**Figure 4**). Interestingly, patients with PE had a significantly higher IgG antibody response against all antigens. Multivariate analysis showed a contribution of peak CRP to the higher IgG response to either RBD, S1S2, or S2 in patients with versus without PE. This contribution was significant for S2 (*p* = 0.013) and almost significant for RBD (*p* = 0.067) and S1S2 (*p* = 0.052). However, the higher IgG response in the PE-positive group remained significant (**Supplemental Data Table 2**). No other contributors were found. By comparing the binding strength data of the SARS-CoV2 antigens with the presence of PE, we found no significant differences (**Supplemental Figure 3**). There was a moderate positive correlation of our *k*_d_ data with the presence of PE for both RBD and NCP (resp. Spearman ρ = 0.33 and Spearman ρ = 0.42).

### Epitope Mapping of Antibody Response Toward RBD

**Figure 5:**
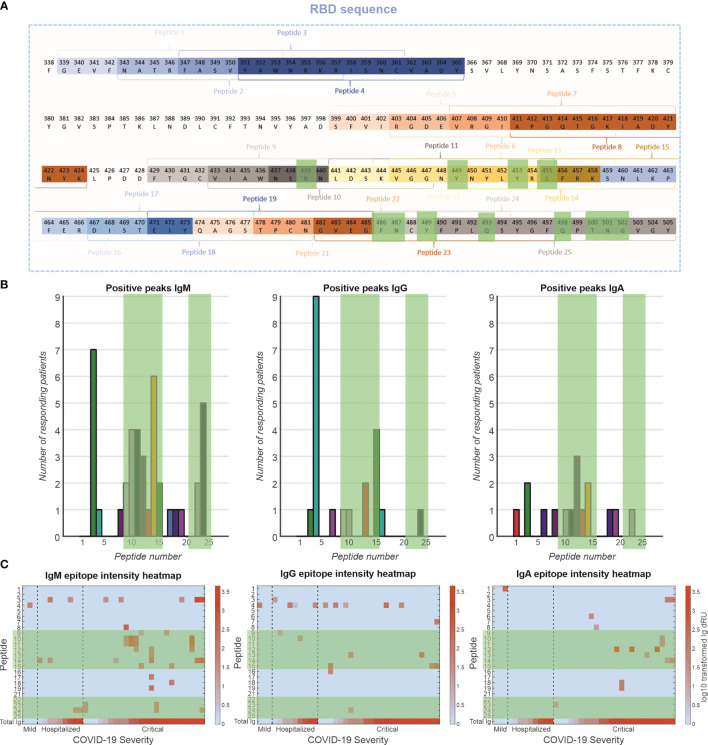
Epitope mapping of antibody response toward RBD. **(A)** A peptide library of 24 peptides was created with four-amino-acid overlap between sequential peptides. These peptides are located at exposed moieties of the RBD and concentrated on the receptor-binding motif (RBM). The known binding motifs between the RBM and ACE2 receptor are highlighted in green. **(B)** Frequency of positive antibody responses toward specific peptides IgM, IgG, and IgA. **(C)** Heatmap of epitope interaction intensity in responder patients. Patients are clustered based on severity and ordered based on total Ig response versus RBD. Heatmap color coding depicts log10 transformed Ig dRU signal.

The antibody response toward specific epitopes on the RBD was characterized using a peptide library spotted on an SPRi sensor (**Figure 5**). Interestingly, of 77 patients measured, only 36 patients (47%) showed antibody responses to the peptide epitopes that spanned the entire receptor-binding motif (RBM) (**Figure 5A**). The antibody responses tended to be directed to peptides encoding amino acid residues that are involved in the interaction with the ACE2 receptor [i.e., peptides 9–15 and 23,24, **Figure 5B** green highlights (23)]. This was particularly prominent for the IgM response, to a lesser extent for the IgG response and least clear for the IgA response. These antibodies are likely neutralizing, that is, blocking the interaction of the RBD of the spike protein with the receptor. Antibodies were also directed to peptides 3 and 4 (IgM and IgG, respectively), which encode amino acids that are not in direct contact with the ACE2 receptor (23), suggesting that these epitopes are exposed and antigenic. We plotted the responders and sorted them based on disease severity (**Figure 5C**). In the IgM response, there seems to be a strong relationship between number of epitopes recognized and disease severity, but not with total Ig response to RBD. In addition, patients with critical disease show robust IgA responses, in contrast to mild or hospitalized patients. Interestingly, there is no relationship between IgG responses to the peptides and disease severity.

## Discussion

COVID-19 patients demonstrate large heterogeneity in disease severity as result of SARS-CoV-2 infection. Literature suggests the humoral immune response is implicated in the disease severity (15, 16, 24, 25), yet the relationship is not fully understood. We have developed a number of assays based on SPRi to more broadly characterize patients’ humoral response and increase our understanding of its contribution to disease progression. We have previously shown SPRi can be used to detect the composition and affinity of the antibody response to SARS-CoV-2 in patients (20, 21). Most studies are performed with commercial ELISAs or immunoassays, and per isotype, a test has to be run. In contrast, the high-throughput SPRi platform is able to measure all three isotypes of immunoglobulins to viral antigen in 96 sera simultaneously. Furthermore, most studies do not include sera from nonhospitalized patients with mild disease. Our study compares three patient groups: nonhospitalized mild disease, moderate disease (hospitalized), and critically ill patients.

In this study serum samples from 76 SARS-CoV-2 patients were analyzed with SPRi using the viral antigens NCP, S1S2, S1, and RBD. For all antigens, significantly higher titers were found in the patients with hospitalized or critical disease severity versus the patients with mild disease. The data further show significant differences between the categories critical, hospitalized, and mild for IgM, IgG, and IgA isotypes. Our results confirm similar findings as reported in other serological studies on SARS-CoV-2 patient cohorts (19, 26–29). In addition to measuring the immune response, SPRi enables us to measure the strength of antibody binding. Recently, we demonstrated that the off-rate (*k*_d_) correlated well with the affinity equilibrium constant (*K*_D_) and as such can be used to rank the antibody response in terms of binding strength (28). Remarkably, patients with critical disease had significantly lower strength of binding antibodies to the RBD in comparison to patients with mild disease, and low-strength antibodies to NCP were seen in the hospitalized patients and patients with critical disease only. This contrasted with binding strength data toward S1S2 and S2, which show higher binding strength toward hospitalized and critical versus mild. An important consideration here, however, is that the patients with mild diseases have rather small antibody responses, making affinity measurements less precise. When we compared the data between critical and hospitalized patients only, we saw that affinity toward all antigens was lower with increasing disease severity. Thus, although in critically ill patients more antibodies to RBD, S1S2, S2, and NCP are produced than in hospitalized patients, their binding strength is lower. As the affinity of the RBD domain for ACE2 receptor is very high [~10 nM (30)], this lower affinity is likely to have severe consequences for the effective neutralization, potentially resulting in more severe disease (30–32). Multivariate analysis showed that the found differences between classes had a contribution of the amount of IgG (RBD, S1S2, or S2) and DPO (S1S2, S2). The *k*_d_ is experimentally independent on the *R*_max_, and therefore on the IgG, the found correlation indicates a clinical relationship. This confirms the finding that the more critical groups with higher IgG responses have larger *k*_d_, showing a relation between those two parameters. The fact that DPO contributed to the *k*_d_ has to be regarded with consciousness as the samples from the group with mild disease had longer DPO than the other groups. As stated previously, this group had also small antibody responses, making the affinity measurements less precise.

We found significant correlation between d-dimer levels and antibody response. In addition, the patients with PE showed higher IgG antibodies to all four tested viral antigens in comparison to the patients without PE. Coagulation pathways and immune system are strictly linked as a physiologic response to contain inflammatory activity at the site of the injury. However, as shown in acute respiratory distress syndrome, pulmonary inflammation-induced coagulopathy may aggravate lung injury and as such contribute to the disease (33). In COVID-19 patients, the hypercoagulable state is reflected by higher levels of d-dimer, fibrinogen, and fibrinogen degradation products (34) and is thought to be exacerbated by a cytokine storm that may be driven among others by activation of macrophages by immune complexes (15). Interestingly, a subgroup of patients with critical disease showed significantly higher immune responses to RBD, S1S2, and S2 antigens, while this subgroup also had significantly higher d-dimer, LDH, and CRP values. These biochemical parameters belong to the biomarkers that are associated with severe and possibly fatal COVID-19 (35).

To elucidate the humoral response in more detail, we developed a peptide library spanning the RBD and analyzed interactions with patients’ sera using SPRi. Our cohort shows a subgroup of patients with antibodies that respond to peptides in the RBD library (47%), equally divided over the severity groups. This could be a limitation of using linear peptides and contrasts with previous research (36). It suggests a subgroup of patients recognizes only conformations epitopes, which should be compared in future work. In patients with antibodies against the peptides, a targeting was observed toward peptides that are associated with ACE2 binding separated over IgM, IgG, and IgA isotypes, indicating an enrichment in potentially neutralization antibodies.

We found an increase in peptide epitopes for IgM isotype in patients with critical disease versus hospitalized patients and patients with mild disease, indicating a broad humoral response. However, we did not find a similar pattern in IgG epitopes. This might indicate an ineffective maturation in the important neutralization epitopes. Furthermore, we found binding of IgA only in critically ill patients supporting an association of prolonged IgA response with unfavorable clinical outcomes (36).

We generally observed a stronger focus of IgM isotype antibodies than IgG or IgA toward the RBD epitopes. As we are looking at unfiltered patient sera, it is possible that the isotypes are competing for binding spots on the peptides. Steric hindrance effects and higher avidity of IgM isotype could potentially skew our data. However, contradicting this, we do not see similar effects between the larger IgA and smaller IgG isotypes.

Men appear to be more at risk for severe disease, worse outcome, and death (37). However, in our cohort, the antibody response of men and women was equal. Similarly, the binding strength of the antibodies in men and women with critical disease did not differ. Only in the hospitalized group, the binding strength of anti-RBD antibodies in male was lower than that in women. This indicates that the primary antibody response between male and female is not solely responsible for the difference in disease severity. It is possible that downstream sex differences in innate immune system responsiveness, for instance, the complement system, might play a critical role (37–39).

The SPRi assays allowed us to characterize the details of the humoral response, including isotype, affinity, and epitopes on a single platform. We applied this to characterize COVID-19 patients; however, this could be applied to any infectious disease. We showed a strong correlation between antibody concentration and disease severity. Yet, these antibodies are of reduced affinity, and maturation on neutralization epitopes might be dysfunctional.

We hypothesize that given the increase in *k*_d_ with increasing disease severity of specific antibodies and incomplete isotype switching, in a subset of patients, antibody production and maturation are ineffective, resulting in a polyclonal antibody pool of lower affinity. As a result, neutralization of SARS-CoV2 is less effective, leading to a higher viral load and increased inflammation. Moreover, in an attempt to effectively neutralize the higher viral count, antibody production increases, and serum levels rise. In neutralization assays, higher antibody titers may compensate for lower affinity, but this comes at the expense of antibody-induced side effects, as these antibodies will find abundant targets potentially leading to Fc-mediated immune responses (40), including activation of complement cascades, increased coagulation, and activation of innate immune cells. Together, this contributes to a hyperinflammatory state and might be implicated in the disease severity of COVID-19 patients.

Limitations of this study concern the number of samples distributed over the groups and the heterogeneity in DPO in the groups. The group with patients with mild infection was relatively small, a direct consequence of the fact that the disease was mild and there was no reason to draw blood. Also, the DPO in the mild group was larger than that in the other groups; this means that IgM responses are not expected anymore. Although multivariate analysis showed no contribution of the DPO to the immunoglobulin responses of either isotype, it is probable that the significantly higher IgM response in the hospitalized and critical groups versus the mild group is a direct effect of the DPO. In addition, there are eight samples taken with a DPO under 10 days, meaning that these samples possibly do not show specific IgG antibodies. The higher IgG response to RBD and S1S2 in patients with critical disease versus the hospitalized group was still statistically significant when the samples with a DPO under 10 days were removed (data not shown).

In conclusion, we have developed multiple assays to broadly characterize humoral responses on a single SPRi platform. We observed significantly larger antibody responses to all SARS-CoV-2 antigens with higher disease severity, yet these antibodies show lower binding affinity in patients with critical disease. Furthermore, while patients with critical disease recognize RBD epitopes associated with ACE2 interaction with IgM isotypes, this is reduced in the case for IgG isotypes. This suggests inadequate isotype switching and maturation. We hypothesize patients with critical disease have large, but ineffective humoral responses to SARS-CoV-2 infection. It remains to be elucidated whether this ineffective humoral response is the result of disease severity or an important driver in COVID-19.

## Data Availability Statement

The raw data supporting the conclusions of this article will be made available by the authors, without undue reservation.

## Author Contributions

JH performed the conceptualization, formal analysis, investigation, methodology, software, visualization, writing of the original draft, review, and editing. RS performed conceptualization, methodology, writing of the original draft, review, and editing. MK performed the conceptualization, data curation, formal analysis, investigation, methodology, writing of the original draft, review, and editing. MD performed the formal analysis and investigation. AC was responsible for resources, writing, review, and editing. AB was responsible for resources, writing, review, and editing. JK was responsible for data curation, formal analysis, methodology, resources, writing of the original draft, review, and editing. AM was responsible for data curation, formal analysis, methodology, resources, writing of the original draft, review, and editing. MK was responsible for conceptualization, methodology, project administration, supervision, writing, review, and editing. JP was responsible for formal analysis and methodology. All authors contributed to the article and approved the submitted version.

## Funding

Nederlandse Organisatie voor Wetenschappelijk Onderzoek and ReumaNederland’s unrestricted research grant LL25 and TTW for the William Hunter perspective program. Both funding sources contributed equally to this work.

## Conflict of Interest

Patents: Provisional patent is filed based on the data generated from this work. Provisional title: Strength of Binding of Antibodies Against Infectious Diseases Measured With Evanescent Field Based Biosensors. COVID-19 Patients With High Titers of Low Affinity Antibodies Are Associated With Disease Severity.

The authors declare that the research was conducted in the absence of any commercial or financial relationships that could be construed as a potential conflict of interest.

## Publisher’s Note

All claims expressed in this article are solely those of the authors and do not necessarily represent those of their affiliated organizations, or those of the publisher, the editors and the reviewers. Any product that may be evaluated in this article, or claim that may be made by its manufacturer, is not guaranteed or endorsed by the publisher.
