# Supplementary material for: High Titers of Low Affinity Antibodies in COVID-19 Patients Are Associated With Disease Severity
Source: Front Immunol. 2022 Apr 13;13:867716. doi: 10.3389/fimmu.2022.867716 (PMC9043688; doi:10.3389/fimmu.2022.867716)
Supplement: Supplementary file 1 [file DataSheet_1.docx]

Supplemental Information

Supplemental table 1. Peptide sequences

| 1 | GEVFNATRFASVYAW |
| --- | --- |
| 2 | NATRFASVYAWNRKR |
| 3 | FASVYAWNRKRI SNC |
| 4 | YAWNRKRISNCVA |
| 5 | SFVIRGDEVRQIAPG |
| 6 | RGDEVRQIAPGQTGK |
| 7 | VRQIAPGQTGKIADY |
| 8 | APGQTGKIADYNYK |
| 9 | FTGCVIAWNSNNLDS |
| 10 | VIAWNSNNLDSKVGG |
| 11 | NSNNLDSKVGGNYNY |
| 12 | LDSKVGGNYNYLYRL |
| 13 | VGGNYNYLYRLFRK S |
| 14 | YNYLYRLFRKSNLKP |
| 15 | LFRKSNLKPFERDIS |
| 16 | SNLKPFERDISTEIY |
| 17 | PFERDISTEIYQAGS |
| 18 | DISTEIYQAGSTPCN |
| 19 | EIYQAGSTPCNGVEG |
| 21 | QAGSTPCNGVEGFNCY |
| 22 | TPCNGVEGFNCYFPLQS |
| 23 | GVEGFNCYFPLQSYGF |
| 24 | FNCYFPLQSYGFQPTN |
| 25 | FPLQSYGFQPTNGVG |
| 26 | SYGFQPTNGVGYQPY |
| 27 | QPTNGVGYQPYRVVV |
| 28 | GVGYQPYRVVVLSFE |

Supplemental table 2. Contribution of the peak CRP to IgG response.

|  | Mean difference PE+ vs PE - | After adjustment for peak CRP |
| --- | --- | --- |
|  |  |  |
| RBD IgG | 1289 (p=0.001) | 916 (p=0.03) |
| S1S2 IgG | 357 (p<0.001) | 255 (p=0.02) |
| S2 IgG | 1097 (p <0.001) | 706 (p=0.028) |

Given are the differences in mean IgG between the PE positive and PE negative group and the differences in mean IgG between the PE positive and PE negative group after correction for the contribution of the peak CRP values. After correction the difference between the two groups are smaller but still statistically significant. Peak CRP did not contribute to the IgG NCP response.


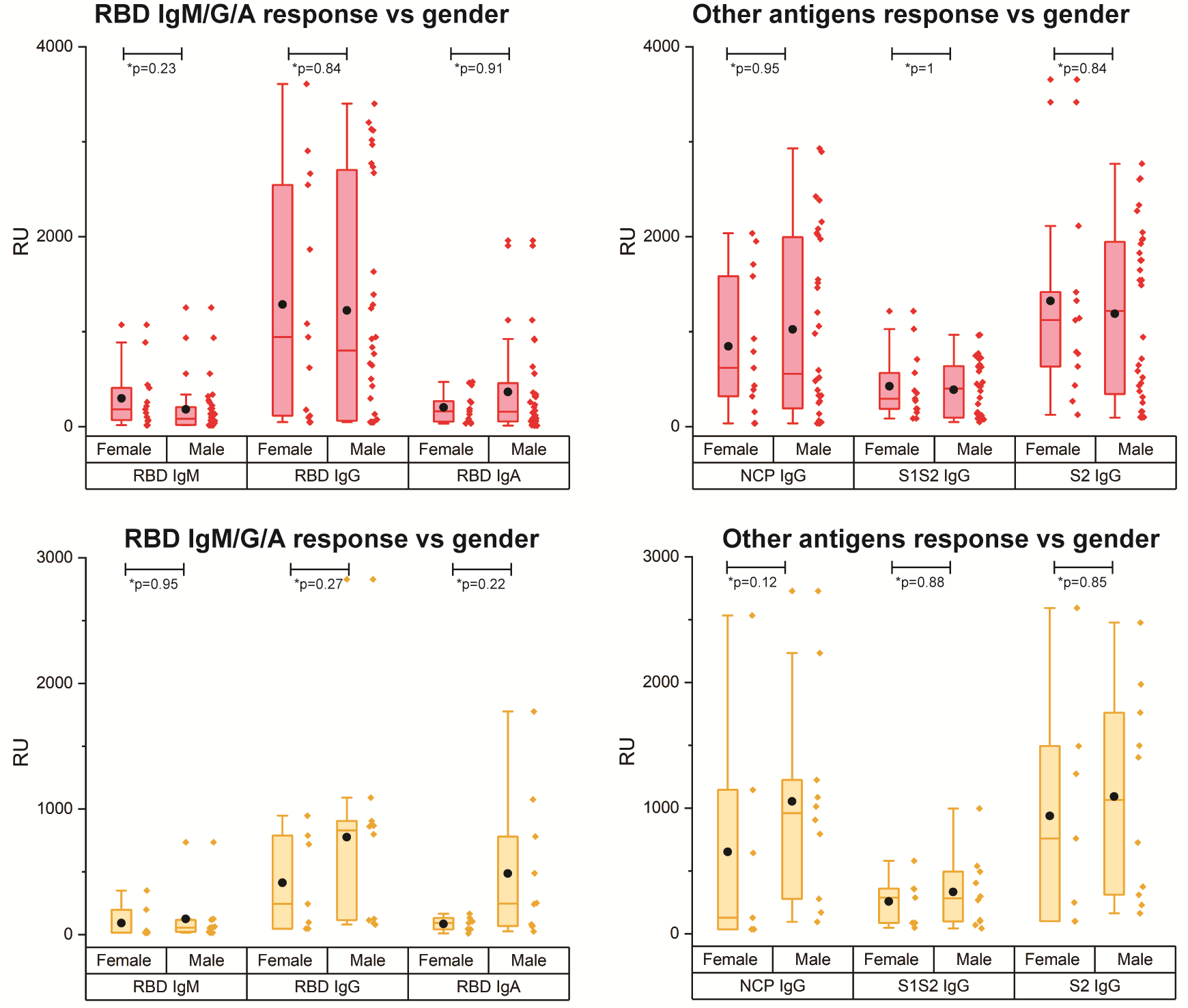


**Fig. S1: Total immune response and the correlation with gender.** The red are the boxplots representing the critical patients and in yellow the hospitalized patients., There were no significant differences between female and male patients for total immune response. The boxplots represent the median, p25 and p75 values and the black dot the mean SPRi RU value (significance tested with Mann-Whitney U test).

**Fig. S2: Binding strength and the correlation with gender.** The red are the boxplots representing the critical patients and in yellow the hospitalized patients. As can be seen, there is a significant difference in the hospitalized patients group for female and male for RBD . Others were non-significant. The boxplots represent the median, p25 and p75 values and the black dot the mean SPRi RU value (significance tested with Mann-Whitney U test).


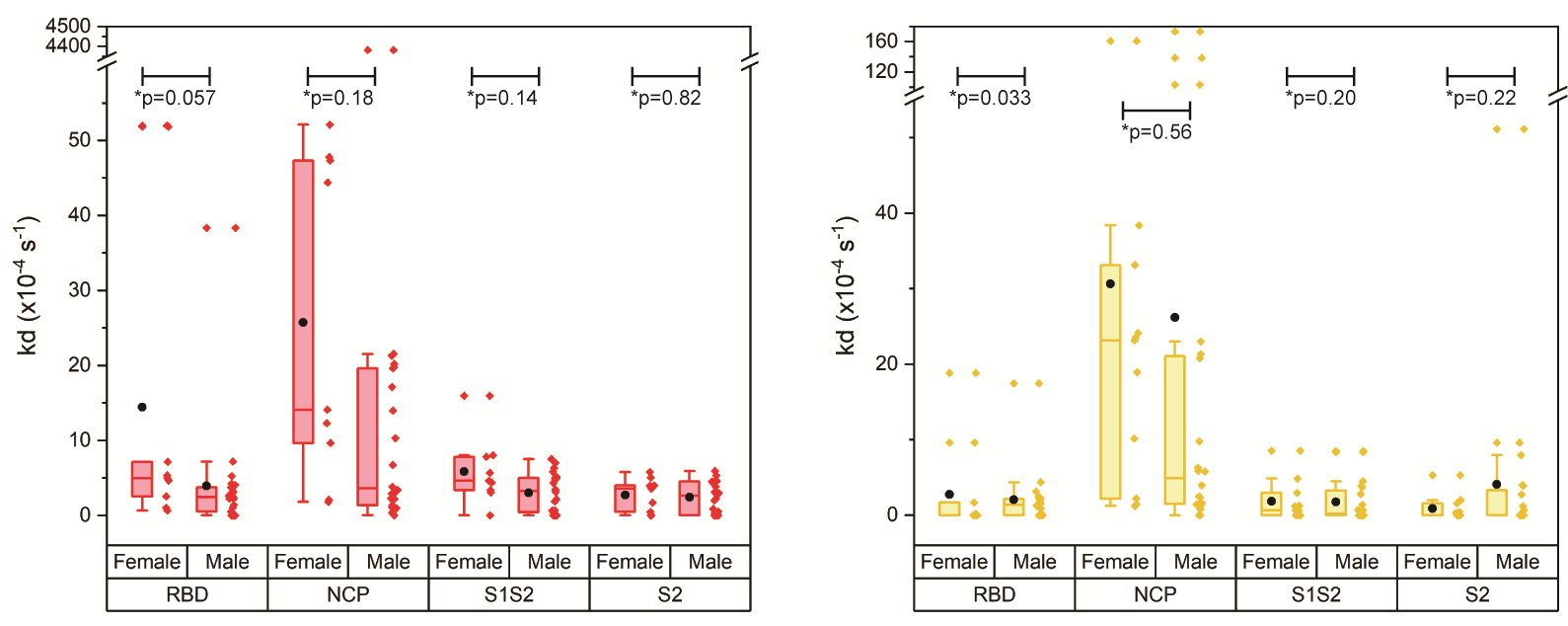

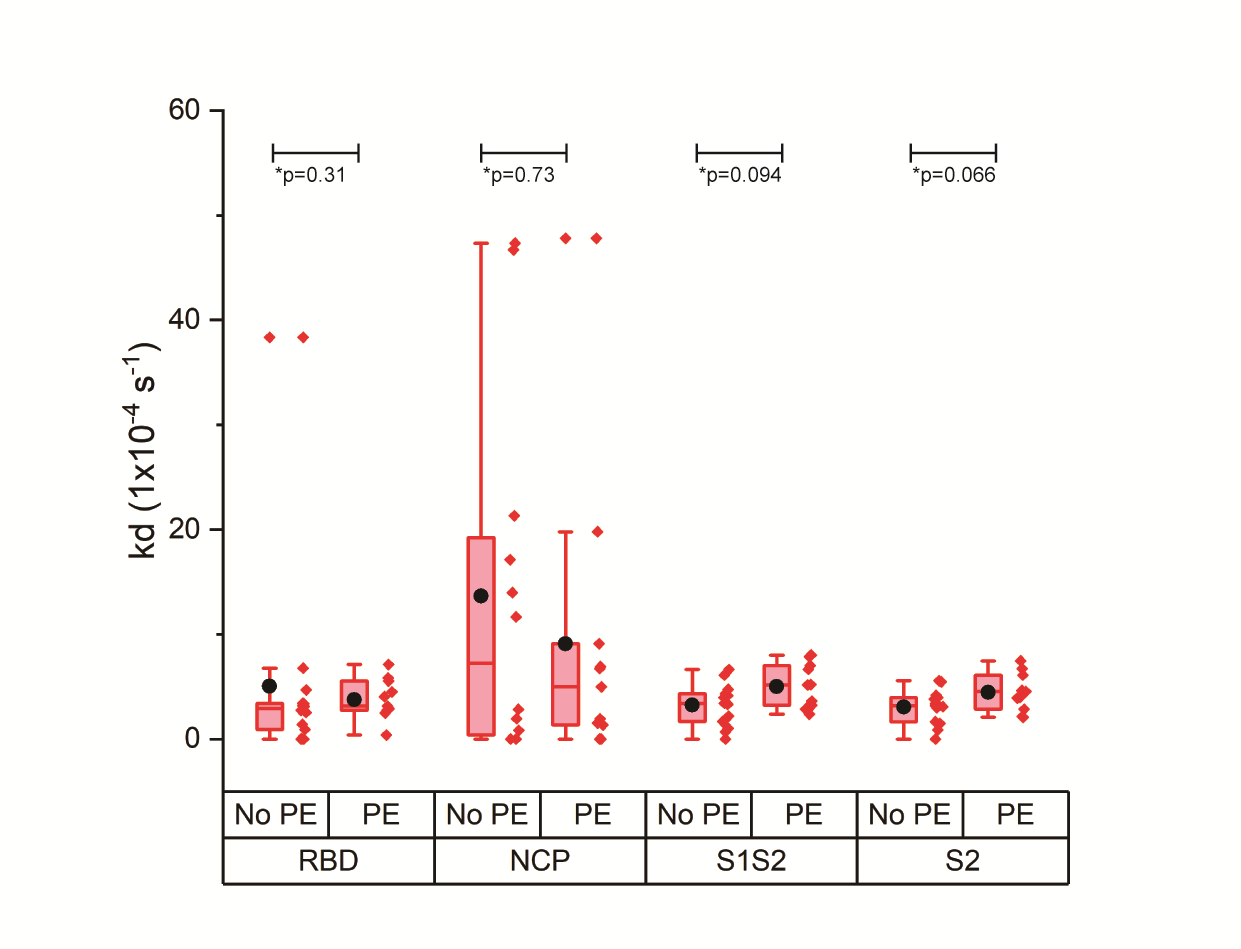


**Fig. S3: Comparison of binding strength data versus occurrence of PE**. Boxplot represents the critical patients. There was no significant difference, determined by Mann-Whitney U test. The boxplots represent the median, p25 and p75 values and the black dot the mean SPRi RU value.


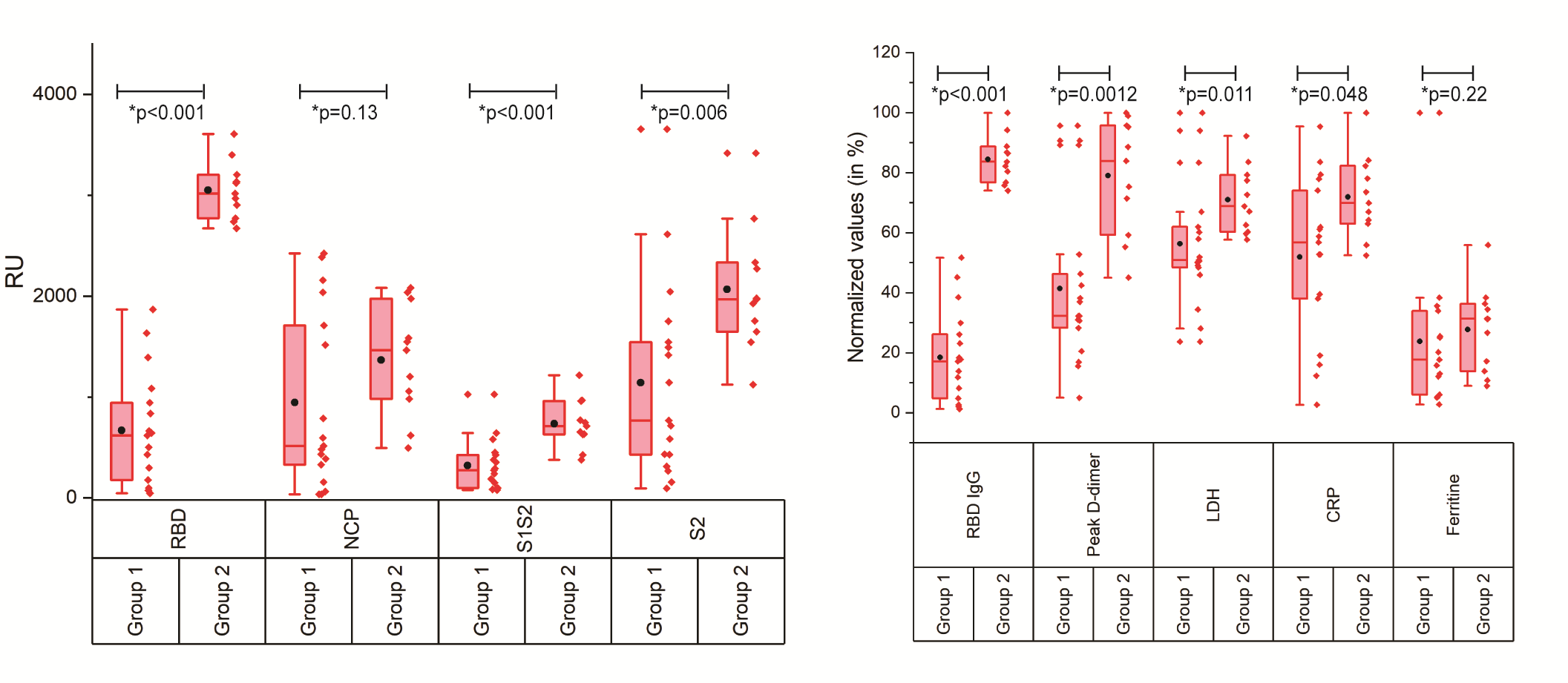


**Fig. S4. Subgroup of RBD IgG for critical patients tested for other IgGs and for laboratory results.** On the left, the subgroup for RBD IgG, NCP, S1S2 and S2. Only NCP did not show a significant difference. On the right, the subgroup was tested for several laboratory results. The data was normalized to percentage, where the highest value per laboratory result corresponds with 100%, Peak D-dimer, LDH and CRP showed a significant difference. The boxplots represents the median, p25 and p75 values and the black dot the mean SPRi RU value (significance tested with Mann-Whitney U test).
